# Clinical Strategies to Improve the Accuracy of Articulating Paper for Detecting Occlusal Contact Points in Adults with Natural Dentitions

**DOI:** 10.3390/diagnostics16101450

**Published:** 2026-05-10

**Authors:** Bernat Rovira-Lastra, Sanaa ElOtmani-Sabiri, Mireia Ustrell-Barral, Laura Khoury-Ribas, Jordi Martinez-Gomis

**Affiliations:** 1 Department of Odontostomatology, School of Dentistry, Faculty of Medicine and Health Sciences, University of Barcelona, 08907 L’Hospitalet, Catalonia, Spain; brovira@ub.edu (B.R.-L.); saelotmani2@gmail.com (S.E.-S.); mireiaustrell@gmail.com (M.U.-B.); laura.khoury.ribas@ub.edu (L.K.-R.); 2 Oral Health and Masticatory System Group, Bellvitge Biomedical Research Institute (IDIBELL), 08907 L’Hospitalet, Catalonia, Spain

**Keywords:** articulating paper, dental occlusion, occlusal registration, reliability, reproducibility of results

## Abstract

**Background/Objectives**: This clinical study assessed the validity of articulating paper for detecting occlusal contacts points, including examining the effects of clinical technique, paper thickness, and the arch. **Methods**: This cross-sectional test–retest study included 32 adults with natural dentitions. Four occlusal registrations were obtained from each participant using articulating paper with a thickness of 100 or 200 μm, applying one of two different clinical techniques (holding in place or pulling with forceps at the intercuspal position), and scanning the occlusal surfaces of their mandibular and maxillary arches. Silicone registrations were obtained and used as the reference standard. Mandibular and maxillary images were scaled and calibrated spatially, and two new images were created based on the sum or the areas of coincidence between the mandibular and maxillary occlusal scheme. Occlusal contact points on the right posterior teeth were analyzed using computer software. **Results**: The articulating paper produced true-positive contacts in 81% and false-positive contacts in 15%, regardless of the method used. Considering occlusal contact when marks matched on both arches accounted for 2.0% of false-positive contact points. General linear models with repeated measures revealed that the mandibular arch offered a higher true-positive percentage than the maxillary arch, that the 100 µm-thick paper produced higher false-positive contacts (20.6%) than the 200 µm-thick paper (9.4%), and that the pulling technique had no significant effect. **Conclusions**: Articulating paper offers good validity when detecting occlusal contact points and can be improved by using 200 µm articulating paper and exploring both arches.

## 1. Introduction

During restorative and prosthetic treatments, clinicians frequently need to adjust the patient’s occlusion intraorally to ensure comfort and proper oral function [1,2]. Because dental occlusion is a key determinant of masticatory performance, the clinician may also need to analyze occlusion in patients with natural dentition during routine practice [3,4,5]. Consequently, occlusal analysis systems must meet the minimum accuracy standards for detecting, quantifying, and localizing occlusal contacts [6,7]. Among the available systems, silicone occlusal registrations scanned with an optical source and analyzed using image-processing software provide the highest reliability and validity for determining the occlusal contact area and precisely locating occlusal contacts [3,8,9,10,11,12]. Digital systems, such as the T-Scan and intraoral scanners, can provide quantitative and temporal occlusal data that can be easily transferred to the dental laboratory [7,13,14,15,16,17,18,19,20,21,22]. Nevertheless, despite the emergence of digital occlusal analysis systems, articulating paper remains the most widely used method for occlusal assessment in clinical settings due to its low cost and ability to rapidly identify contact locations [17,18,19,23,24,25,26].

For clinical assessment of static occlusion with articulating paper, the clinician dries the occlusal surfaces, places a strip of a selected thickness into forceps, and instructs the patient to close into maximum intercuspation several times while the paper is held in place [23]. The color transferred from the articulating paper to the occlusal surface is assumed to be accurate, with each mark representing a true occlusal contact, thereby allowing intraoral visualization by the clinician. However, each step in this procedure may introduce variability and error, potentially affecting the accuracy of occlusal analysis, particularly factors such as the patient’s ability to achieve the correct mandibular position and to exert sufficient occlusal force. Moreover, the choice of articulating paper thickness, articulating paper placement, and interpretation of color marks are operator-dependent [1,13,24,27,28,29]. Variations in the clinical technique, such as the two-step method or the parallel cut method (without complete separation of the paper), may offer certain advantages [30,31]. However, it remains unclear whether pulling the articulating paper during clenching improves the accuracy of occlusal contact localization. Similarly, the preferred arch for visual examination is not well established: whereas the mandibular arch is more accessible to the clinician, the maxillary arch is more stable and less influenced by the saliva [1,32].

The accuracy of an occlusal analysis system can be evaluated in terms of criterion validity—defined as agreement with a reference standard—as well as through test–retest and inter-rater reliability, which reflect the consistency of measurements over time and across different clinicians [33]. The accuracy of articulating paper in measuring the occlusal contact area and locating occlusal contacts has been previously investigated [11]. However, in routine clinical practice, clinicians may be more interested in detecting and locating the occlusal points than with delineating contact boundaries or measuring contact area [9,34].

The purpose of this clinical study was to determine the validity of articulating paper for locating occlusal contact points. Additionally, the study assessed whether different articulating paper-based techniques—specifically paper thickness, the pulling technique, and the arch examined—affect validity as well as test–retest and inter-rater reliability. Finally, the percentage of false-positive marks on cusp tips was explored. The null hypothesis was that the different techniques would demonstrate similar validity for locating occlusal contact points.

## 2. Materials and Methods

This cross-sectional study was conducted on 35 dental students aged 18–45 years, who had healthy dentitions with at least 24 natural teeth and no edentulous spaces. Individuals with extensive dental restorations, dental prostheses, severe malocclusions, ongoing orthodontic treatment, or orofacial pain were excluded. All participants had taken part in previous investigations and had signed a written informed consent form approved by the Ethics Committee of Barcelona University Dental Hospital (Ref. 11/2020) [11,35]. All procedures were conducted according to the principles of the Helsinki Declaration. Reporting follows the STROBE guidelines.

All clinical procedures were performed by a single operator with more than 10 years of clinical experience (B.R.-L.). The study participants were seated in a dental chair oriented with the Frankfort plane parallel to the floor, and the operator ensured no debris was present on the occlusal surfaces of the teeth. Next, a polyvinyl siloxane occlusal registration material (Occlufast Rock; Zhermack, Badia Polesine, Italy) was applied to the mandibular teeth and participants were asked to occlude with maximum force at the maximum intercuspation position for 1 min [36]. The occlusal registration was removed, carefully trimmed, scanned using a transparency adapter on a flatbed scanner (HP Scanjet G4050; Hewlett Packard, Palo Alto, CA, USA), and saved in JPEG format [5].

For each participant, four occlusal registrations were obtained using articulating paper. The operator positioned either 100 µm articulating paper (Blue, Progress 100 µm; Bausch; Cologne, Germany) or 200 µm articulating paper (Blue, Articulating Paper BK01; Bausch) on each hemiarch, held with two Miller forceps (Miller; Carl Martin; Solingen, Germany). Participants were instructed to close firmly into maximum intercuspation position three times and care was taken to ensure proper execution. Two clinical techniques were applied during closure. In the conventional “passive” technique, the operator held the forceps stationary without applying additional movement. In the “pulling” or “active” technique, the operator attempted to pull the forceps out from between the occluding teeth as the participant closed into maximal intercuspal position. Before placing the paper, cheek retractors (Spandex; Hager Worldwide; Hickory, NC, USA) were inserted, saliva was suctioned with a standard saliva ejector (Monoart; Euronda), and the occlusal surfaces were air dried with an air-syringe. After removing the articulating paper, the marks on the maxillary and mandibular arches were scanned (TRIOS 3; 3Shape A/S; Copenhagen, Denmark) and the teeth were cleaned with a cotton roll and nylon brush (Proclinic; Stoddard Manufacturing Co; Hertfordshire, UK) to remove any occlusal marks.

The sequence of occlusal recordings for each technique was randomized based on permuted blocks using web-based software (http://www.randomization.com). This resulted in approximately half of the participants being assigned to each sequence. To determine the reliability of the research method and the reproducibility of the occlusal methods for detecting occlusal contact points, all participants repeated the clinical procedure 2 weeks after the initial procedures.

**Figure 1:**
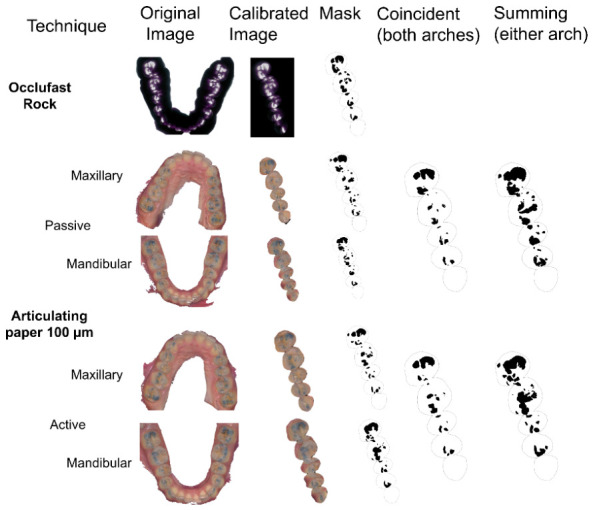
Image-processing workflow for occlusal records. The original articulating paper and Occlufast Rock images were calibrated and converted into 8-bit mask images. Additional 8-bit masks were generated, including “coincident” and “sum of occlusal contacts” masks, by combining the maxillary and mandibular arches using the “Add” and “AND” functions of the FIJI Image Calculator. This procedure was performed for each articulating paper thickness and for each clinical technique.

Half of the occlusal registration images were calibrated with a reference image obtained with an articulating paper of the maxillary arch, with the other half calibrated using the mandibular arch; all used a FIJI software program (version 1.54p; ImageJ; National Institutes of Health; Bethesda, MD, USA) [11]. The occlusal perimeter of the premolars and molars of the right side of the reference image was selected and saved as the region of interest. For images calibrated with the maxillary arch, the canine was included in the occlusal perimeter. All color images were transformed using multiple points of equivalence on a scale-calibrated reference image with a “transform” plugin, applying a similarity class transformation with the least-squares method. Each color image was then converted to a grayscale 8-bit format showing the occlusal contacts as black marks (Figure 1). Occlufast Rock images were converted to 8 bits with a gray-level threshold value of 146 to generate an image with contact areas at interocclusal distances of 100 μm. The blue marks from articulating paper registrations were converted to grayscale using the CIELab color space with threshold values of 1 to 255, 0 to 255, and 0 to 130 for L*, a*, and b*, respectively. When converting the colored images to grayscale, the color image was added as an overlay with 90% transparency to correct the occlusal mark boundaries with the FIJI brush options if needed. For images that used the maxillary arch as reference, occlusal contact on the maxillary canine was only considered when it contacted against a mandibular premolar.

To compensate for image deformation and errors produced when converting to two-dimensional images, all articulating paper masks were transformed using the “TurboReg” plugin and the Occlufast Rock mask for reference. The mandibular and maxillary masks were combined using the commands “add” or “AND” of the FIJI Image Calculator to create new masks that represented the occlusal contacts in both arches (coincident) or either arch (summing) (Figure 1).

**Figure 2:**
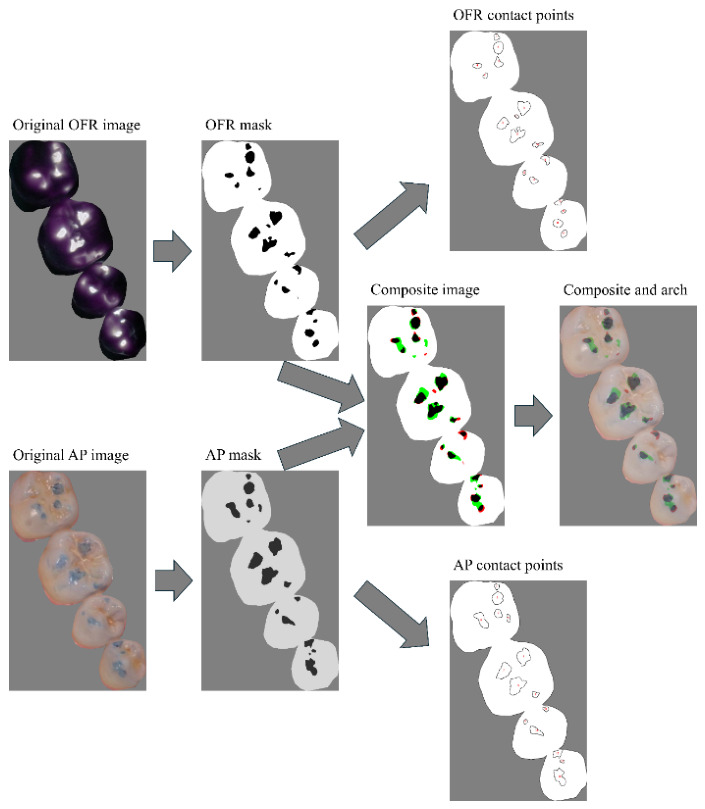
Image-processing protocol for quantifying occlusal contact points and determining true- and false-positive contacts. Original articulating paper (AP) and Occlufast Rock (OFR) images were calibrated and converted into 8-bit mask images. A composite image was then generated by merging the AP and OFR masks. A true-positive contact was defined as an Occlufast contact containing at least one black pixel, whereas a false-positive contact was defined as an articulating paper mark composed entirely of green pixels. Additionally, false-positive contacts located on cusp tips were identified and quantified using the composite image overlaid with the dental arch. The number of occlusal contact points in each mask was determined using the “Analyze Particles” command in FIJI.

The number of occlusal contact points per mask was determined by considering a contact point to be at least 100 pixels squared (approximately 0.5 mm^2^) and calculated with the “analyze particles” command once a threshold was set (Figure 2). A composite was created with each articulating paper mask and overlaid with the Occlufast Rock mask using the “merge canals” function. This color image consisted of black and white areas as true-positive and true-negative contacts, respectively, and of green and red areas as false-positive and false-negative contacts, respectively (Figure 2). The Occlufast Rock contact points that had at least one black pixel in the composite image were considered a true-positive contact point, whereas contact points from the articulating paper that had no black pixels were considered a false-positive contact point. The composite image was overlaid with the occlusal arch to count the number of false positives on cusp tips. Among the false-positive points (green areas), those on cusp tips were classified as cusp-tip false positives.

Image processing and data analysis from the test and retest sessions were performed by a single researcher (J.M.-G.) with over 20 years of clinical experience. To assess the inter-rater reliability of the image processing and interpretation of occlusal contact points, another researcher (S.E.-S.) assessed the number of occlusal points in each occlusal registration.

The sample size calculation was based on the primary outcome (sensitivity, defined as the proportion of true-positive occlusal contact points). Assuming a sensitivity of 70% [11], with a significance level (α) of 0.05 and a precision (d) of 7.5%, the required sample size was estimated using the formula n = (Z^2^ × Se × (1 − Se))/d^2^. This resulted in a requirement of 143 occlusal contact points, corresponding to approximately 14 participants, given an average of 20 occlusal contacts per individual at the intercuspal position (10 per side) [34]. To account for the clustering of occlusal contact points within participants (design effect) and to ensure robustness, the sample size was increased to 32 participants.

The Shapiro–Wilks test was used to confirm the normality of the distribution for the number of occlusal contact points. Test–retest reliability for determining occlusal contact points and the percentages of true positives and false positives in localizing occlusal contact points was assessed by the intra-class correlation coefficient (ICC) for single measurements, using a two-way random effects model and absolute agreement. The mean value from the two measurement sessions was calculated and subsequently used for the descriptive analysis of occlusal contact points and cusp-tip false-positive findings. Three general linear models with repeated measures (GLM-RM) were performed to understand the effect of several factors on the percentages of true-positive and false-positive contact points and on the number of cusp-tip false positives, as dependent variables. The within-subject factors considered in each GLM-RM were the session (1 = test; 2 = retest), articulating paper thickness (1 = 100 µm; 2 = 200 µm), pulling technique (1 = passive; 2 = active), and dental arches (1 = mandibular; 2 = maxillary; 3 = Coincide; 4 = Either arch), except for the third GLM-RM (1 = mandibular; 2 = maxillary). Data are reported as means with ranges or 95% confidence intervals (CI). The statistics were analyzed with IBM SPSS Version 30 for Windows (IBM Corp, Armonk, NY, USA) and a *p*-value of <0.05 was considered to be statistically significant.

## 3. Results

Among the 35 individuals examined, 3 were excluded (poor quality images) and the 32 remaining participants (25 women and 7 men) had a mean age of 24.5 years (95%CI, 23.1–25.9) and a mean of 28.5 teeth (range, 25–32). The number of occlusal contact points determined by each technique and both the inter-rater and test–retest reliability are shown in Table 1. The mean number of occlusal points ranged from 11 to 13 using Occlufast Rock and the articulating paper when examining only one arch. The ICC values for the inter-rater and test–retest reliability when using Occlufast Rock to determine occlusal points were 0.999 and 0.976, respectively; the corresponding values when using articulating paper were in the ranges 0.56–0.88 and 0.54–0.82.

The first GLM-RM analysis showed that the overall mean percentage of true-positive occlusal points was 81% and that this value was significantly dependent on the arch examined (*p* < 0.001) (Table 2). Approximately 5% more true contacts were detected when the dentist examined the mandibular arch compared with the maxillary arch (*p* < 0.001). Paper thickness, session, and clinical technique had no significant effect on the percentage of true positives.

The second GLM-RM analysis revealed that the percentage of false-positive occlusal points depended on the arch examined (*p* < 0.001) and the thickness of the articulating paper (*p* < 0.001) (Table 2). The percentages of false positives were 9.4% using the 200 µm paper and 20.6% using the 100 µm paper. A similar percentage of false positives, 15%, was found when examining the mandibular and maxillary arches, but this decreased to 2% when applying the criterion that the blue mark should coincide on both arches to be considered a point of occlusion. The mean number of false positives located in a prominent trait (cusp tip) was 1.2 per hemiarch (Table 2). Regardless of the paper thickness, the pulling technique, and the arch examined, a mean of 65.9% (95%CI, 59.6–72.3%) of false positives were located on the cusp tips.

The third GLM-RM analysis showed that session (*p* = 0.788), arch examined (*p* = 0.486), and clinical technique (*p* = 0.759) did not affect the number of false positives on a cusp tip. However, using the 200 µm-thick paper produced fewer false positives (0.61; 95%CI, 0.4–0.9) than using the 100 µm-thick paper (1.75; 95%CI, 1.5–2.0) (*p* < 0.001).

Regardless of the method used, the ICC values for test–retest reliability of the percentages of true positives, false positives, and cusp-tip false positives were 0.571 (95%CI; 0.28–0.77; *p* < 0.001), 0.615 (95%CI; 0.35–0.79; *p* < 0.001), and 0.247 (CI95% −0.12–0.55; *p* = 0.088), respectively.

## 4. Discussion

This study showed that articulating paper could detect 81% of occlusal contacts points in the posterior region, regardless of the method used. This percentage of true-positive occlusal points can be increased when the dentist examines only the mandibular arch, with blue marks from articulating paper being better preserved compared with the maxillary arch. Examining the mandibular arch through direct inspection is also easier for the dentist. Therefore, the null hypothesis that different techniques would have similar validity for locating occlusal contact points was rejected.

When using articulating paper and any method, 15% of occlusal contacts were color marks that did not correspond to true occlusal contacts. However, very few false-positive contacts were observed when both the mandibular and maxillary arches were examined and occlusal contact was only considered present when color marks matched in both arches. Interestingly, two-thirds of false-positive contacts were blue marks on a cusp tip, corresponding to a mean of 1.2 cusp-tip false positives per sextant. These color marks likely resulted from rubbing the articulating paper on the cusp of mandibular or maxillary teeth close to true occlusal contacts. Therefore, a color mark located on a cusp tip that does not match with the antagonist tooth should be considered a false positive, particularly in occlusal adjustment procedures.

In general, it is expected that the number of marks increases and the occlusal area widens as the thickness of articulating paper increases [11,23,24,27,37,38]. However, articulating papers of 100 µm or 200 µm thicknesses have been observed to show similar numbers of occlusal contact points and similar occlusal contact areas [11,37]. The present study shows that using the 200 µm paper achieved not only fewer false-positive contact points than the 100 µm paper but also fewer false-positive marks on the cusp tip. Therefore, the 200 µm articulating paper may offers superior accuracy for both locating occlusal contacts and detecting occlusal points [11]. However, these results cannot be extrapolated to articulating papers thinner than 100 µm, and future studies should evaluate the accuracy of articulating papers and films with low-to-medium thicknesses (8–40 µm).

The present results suggest that applying the pulling technique offered neither a clear benefit nor an inconvenience when detecting occlusal contact points. Other aspects of the clinical technique may be more relevant, such as placing the paper appropriately between arches, using adequate forceps, saliva control, verifying that the patient is able to close at the intercuspal position, not dragging the articulating paper on tooth surfaces, and avoiding repeated use of the same paper (especially when fragmented, perforated, or the ink has been lost or used) [13,27,39,40]. However, to the authors’ knowledge, no study has systematically assessed the relevance of each aspect of the clinical technique on the validity and reproducibility of detecting occlusal contact points. This gap highlights the need for further research aimed at developing clinical guidelines for general practitioners.

Articulating paper provided moderate to good inter-rater reliability for detecting occlusal contact points, regardless of the method used, with similar figures for measuring the occlusal contact area [11]. This emphasizes the clinical subjectivity when interpreting occlusal records by examining intraoral marks as part of the occlusal analysis [1,13,24]. Although the application of deep learning may improve the efficiency of detecting occlusal contacts [35], the chromatic intensity of the blue mark can also provide information about occlusal force. In theory, true contacts are expected to present as a central light-blue area surrounded by a darker-blue halo, reflecting near-contact or non-contact regions [24,31]. However, this chromatic pattern was not observed in most occlusal contacts in the present study. Other chromatic characteristics may provide additional information regarding false-positive contacts or variations in occlusal force, potentially enabling future support through artificial intelligence. Another way to improve interpretation would be to ask the patient what teeth first contact each other in the intercuspal position, especially when adjusting a restoration for optimal occlusion [13,27].

The test–retest reliability of the percentage of true and false positives in detecting the occlusal contact points was moderate, and these values indicate that intra-individual variability is comparable in magnitude to inter-individual variability. By contrast, the low ICC value for the percentage of cusp-tip false positives indicates high intra-individual variability, which might not be typical of a delimited patient group. Rather than not allowing cusp tips to rub, clinicians should verify whether the color mark on a cusp tip has a matching point on the antagonist tooth.

This study included all procedures for detecting occlusal points in clinical practice. However, several limitations should be acknowledged. First, only posterior teeth were assessed. The inclusion of anterior might reduce accuracy, as occlusal contact detection in incisors and canines is more sensitive to small variations in mandibular position during closure to maximum intercuspation. Second, the findings cannot be generalized to patients with extensive restorations, removable or implant-supported prostheses, or occlusal disorders. In addition, the image-processing protocol included manual correction steps performed by the operator, which may have introduced bias due to the subjective interpretation of the blue marks, potentially leading to an overestimation of the accuracy of articulating paper. The sensitivity for locating occlusal contact points, assessed as the percentage of true-positive occlusal points, may be influenced by interindividual variability in the number and distribution of occlusal contacts, which could affect the precision of statistical estimates. Finally, the effects of the clinical technique on the accuracy of the occlusal analysis system evaluated in this study are specific to articulating paper and may not be applicable to digital systems, such as T-Scan, which demonstrate good test–retest reliability and excellent inter-rater reliability, but exhibit higher false-positive rates than conventional systems in the localization of occlusal contacts [8,11,16]. Consequently, future research should include the assessment of anterior occlusal contacts in individuals with natural dentition, as well as patients with different types of prostheses and restorative materials. Further studies should also evaluate digital occlusal analysis systems, such as the T-Scan, Occlusense, ModJaw, and intraoral scanners.

The results of the present study provide evidence to inform several clinical recommendations. Specifically, the use of 200 µm articulating paper appears to be preferable to 100 µm paper; occlusal contact should be considered present when color marks located on cusp tips are observed on both the maxillary and mandibular arches; and the pulling technique does not improve the accuracy of detecting occlusal contact points.

## 5. Conclusions

The use of articulating paper, regardless of method, was able to detect 81% of occlusal contacts points in the posterior region, with a 15% false-positive rate. Two-thirds of the false positives were blue marks located on cusp tips, probably caused by accidentally rubbing the paper against these prominences. Very few false-positive contact points could be observed when an occlusal contact was considered true only if the blue marks matched on both the maxillary and mandibular arches. The use of 200 µm articulating paper resulted in fewer false-positive contacts than 100 µm articulating paper. However, the pulling technique did not improve the accuracy of detecting occlusal contact points.

## Figures and Tables

**Table 1:** Mean occlusal contact points on right posterior teeth with test–retest and inter-rater reliability shown for the different methods used.

Method	Occlusal Contact Points		
	Occlusal Points Mean Number (95%CI)	Inter-Rater Reliability ICC (95%CI)	Test–Retest Reliability ICC (95%CI)
Occlufast Rock	12.9 (11.5–14.2)	0.999 (0.998–0.999)	0.976 (0.951–0.988)
Articulating Paper			
100 µm; passive; mandibular	13.2 (11.9–14.6)	0.877 (0.764–0.938)	0.648 (0.395–0.810)
100 µm; active; mandibular	13.3 (12.1–14.6)	0.634 (0.368–0.803)	0.752 (0.553–0.871)
100 µm; passive; maxillary	12.1 (10.7–13.5)	0.780 (0.592–0.887)	0.662 (0.378–0.826)
100 µm; active; maxillary	12.1 (10.8–13.5)	0.812 (0.643–0.904)	0.698 (0.469–0.839)
100 µm; passive; mandibular AND maxillary	9.1 (7.7–10.4)	0.809 (0.449–0.922)	0.816 (0.657–0.906)
100 µm; active; mandibular AND maxillary	9.2 (8.0–10.5)	0.614 (0.328–0.794)	0.795 (0.623–0.894)
100 µm; passive; mandibular OR maxillary	15.0 (13.5–16.5)	0.842 (0.703–0.919)	0.684 (0.296–0.855)
100 µm; active; mandibular OR maxillary	14.7 (13.5–15.9)	0.614 (0.346–0.790)	0.580 (0.293–0.771)
200 µm; passive; mandibular	11.6 (10.3–13.0)	0.797 (0.624–0.896)	0.726 (0.511–0.856)
200 µm; active; mandibular	11.7 (10.5–12.9)	0.752 (0.550–0.871)	0.680 (0.436–0.830)
200 µm; passive; maxillary	10.5 (9.3–11.8)	0.772 (0.583–0.882)	0.552 (0.253–0.754)
200 µm; active; maxillary	10.7 (9.6–11.8)	0.768 (0.575–0.880)	0.543 (0.250–0.746)
200 µm; passive; mandibular AND maxillary	8.8 (7.8–9.8)	0.558 (0.199–0.771)	0.587 (0.301–0.776)
200 µm; active; mandibular AND maxillary	9.5 (8.5–10.5)	0.617 (0.336–0.795)	0.622 (0.351–0.796)
200 µm; passive; mandibular OR maxillary	12.2 (10.8–13.5)	0.744 (0.538–0.867)	0.702 (0.475–0.842)
200 µm; active; mandibular OR maxillary	11.8 (10.6–13.0)	0.754 (0.553–0.872)	0.630 (0.362–0.801)

95%CI, 95% confidence interval; ICC, intra-class correlation coefficient.

**Table 2:** Measures of test accuracy by session, paper thickness, arch examined, and pulling technique analyzed using general linear models with repeated measures.

		True-Positive Points (%)		False-Positive Points (%)		Cusp-Tip False-Positive Points (*n*)	
Variable	Categories	Mean (95%CI)	Significance	Mean (95%CI)	Significance	Mean (95%CI)	Significance
Session			0.76		0.40		0.79
	Test	81.3% (78–85)		14.5% (11–18)		1.2 (0.9–1.4)	
	Retest	80.7% (77–85)		15.5% (13–19)		1.2 (1.0–1.4)	
Paper Thickness			0.36		<0.001		<0.001
	100 µm	82.0% (78–86)		20.6% (17–24)		1.8 (1.5–2.0)	
	200 µm	80.0% (76–84)		9.4% (6–13)		0.6 (0.4–0.9)	
Arch examined			<0.001		<0.001		0.49
	Mandibular	84.5% (81–87)		15.1% (12–18)		1.1 (0.8–1.4)	
	Maxillary	78.8% (75–83)		14.8% (11–18)		1.2 (1.0–1.5)	
	Coinciding	72.3% (68–77)		2.0% (1–3)			
	Sum	88.5% (86–91)		28.1% (23–33)			
Clinical technique			0.76		0.34		0.76
	Passive	80.0% (76–84)		14.6% (12–18)		1.2 (1.0–1.4)	
	Pulling	82.0% (79–85)		15.4% (12–19)		1.2 (0.9–1.4)	
Overall mean		81.0% (78–84)		15.0% (12–18)		1.2 (1.0–1.4)	

95%CI, 95% confidence interval.

## Data Availability

The data presented in this study are available upon request from the corresponding author. The data are not publicly available due to privacy reasons.
